# EU‐27 Public Opinion on Brexit^†^

**DOI:** 10.1111/jcms.13107

**Published:** 2020-12-04

**Authors:** Stefanie Walter

**Affiliations:** ^1^ University of Zurich Zurich

**Keywords:** Brexit, public opinion, international negotiations, EU‐27, negotiation preferences

## Abstract

Although there has been much interest in British public opinion on Brexit, much less is known about how EU‐27 Europeans view the Brexit negotiations. This is surprising, because Brexit confronts the EU‐27 with difficult choices. Whereas accommodating the UK carries the risk of encouraging further countriesto leave the EU, an uncompromising negotiation stance increases the economic and social costs of Brexit. Using original survey data from 39,000 respondents in all EU‐27 countries collected between the start of the Brexit negotiations and December 2018, this article shows that exposure to the economic risks of Brexit makes respondents more willing to accommodate the UK, whereas a positive opinion of the EU decreases their willingness to compromise. Moreover, many Europeans face an accommodation dilemma that moderates these preferences. Overall, the EU‐27 public unsentimentally supports a Brexit negotiation line that safeguards their own interests best.

With Brexit described as ‘the will of the British people’, research on Brexit‐related public opinion is burgeoning. Many studies have examined voting behaviour in the Brexit referendum (Alabrese *et al.,* 2019; Clarke *et al.,* 2017; Colantone and Stanig, 2018; Goodwin *et al.,* 2018; Henderson *et al.,* 2017; Hobolt, 2016; Vasilopoulou, 2016). Others have tried to identify what kind of Brexit British voters actually want (Hobolt and Leeper, 2017; Renwick *et al.,* 2018; Richards *et al.,* 2018), whether their knowledge about and perceptions of the EU have changed since the 2016 vote (Grynberg *et al.,* 2019) and how Brexit affects the electoral behaviour and public opinion in the UK more generally (Hobolt, 2018; Hobolt *et al.,* 2020). Beyond academia, policy‐makers, journalists, and think tanks have tried to identify what British voters want or not want from the Brexit process and which types of Brexit arrangements might be acceptable to them.

This detailed attention to British public opinion on Brexit is mirrored by a dearth of research on Brexit‐related public opinion in the remaining EU member states. Not even a handful of studies exists (De Vries, 2017; Jurado *et al.,* 2018; Walter 2020a). This is surprising because Brexit is likely to have considerable consequences not just for the British public, but for EU‐27 citizens as well. In economic terms, researchers estimate that the costs of a negotiated, but ‘hard’, Brexit that reimposes considerable trade frictions between the UK and the EU will be about 2.6 per cent of the EU‐27's overall GDP (Chen *et al.,* 2018). Some countries are exposed to much higher costs, most notably Ireland (where 10.1% of GDP is estimated to be at risk), Germany (5.5%) and the Netherlands (4.4%). The fallout from a ‘no deal Brexit’ would be even costlier. It is thus clear that Brexit can have significant negative consequences for voters in the EU‐27. In political terms, Brexit also has consequences for the future of the EU. The exit of one of the Union's biggest members changes the political balance among the member states and opens question such as how to address the loss of British contributions to the EU's budget.

Moreover, Brexit carries risks of political contagion: Brexit may embolden eurosceptics in the remaining member states, potentially leading to further attempts among the EU‐27 to exit the EU (De Vries, 2017; Walter, 2020a). Fears of contagion risks have receded since the 2016 Brexit referendum, as support for the EU has surged and eurosceptic parties have ceased to call for EU exits of their own countries in their agendas (Chopin and Lequesne, 2020; Glencross, 2019). Nonetheless, there is some evidence that a favourable outcome of the Brexit negotiations for the UK may reignite support for an EU exit within the remaining member states, as voters benchmark their own country's prospects within the EU against a positive trajectory of the UK outside the EU (De Vries, 2017, 2018; Walter, 2020a, 2020b). Brexit may thus pose a serious threat for the EU as a whole (Hobolt, 2016; Oliver, 2016), especially at a time when European integration has become a heavily contested issue among European voters and elites (De Wilde and Zürn, 2012; Hobolt and de Vries, 2016; Hooghe and Marks, 2009). In such a context, national electoral considerations have a strong impact on the dynamics of international negotiations (Kleine and Minaudier, 2019; Schneider, 2020).

The consequences of Brexit for citizens of the EU‐27 provide a motivation to look at their Brexit‐related concerns and views about the Brexit negotiations in their own right. However, exploring EU‐27 public opinion also matters because of its potential influence on the Brexit negotiations themselves. Experimental research shows that policy‐makers take public opinion into account when making foreign policy decisions, especially when they fear that the government will pay significant political costs if they fail to heed public opinion (Tomz *et al.,* 2020). 1 These dynamics also apply to decision‐making in the EU Council, where governments are responsive to domestic public opinion (Hagemann *et al.,* 2016; Schneider, 2018). This responsiveness is particularly strong when integration‐related EU‐Council decisions are taken in contexts in which the salience of EU integration in the public sphere is high, such as during the Brexit negotiations (Wratil, 2018). Moreover, the literature on two‐level games in international negotiations notes that voters' preferences can enhance governments’ bargaining power in international negotiations (Caraway *et al.,* 2012; Hug and König, 2002; Schneider and Cederman, 1994).

Because national governments are key actors in the Brexit negotiation process, all of this suggests that public opinion in the EU‐27 member states is likely to play a role in Brexit negotiations. After all, it is the member states who, via an EU Council decision on the adoption of negotiation directives, set the Brexit negotiation mandates for the EU Commission‐led Brexit negotiation team. The member states also have to ratify the outcomes of the Brexit negotiations jointly with the European Parliament. 2 Whereas the withdrawal agreement had to be ratified only by the EU Council and the European Parliament, any agreement about the future relationship between the EU and the UK will have to be ratified by each member state separately, thus additionally involving national parliaments. 3 Thus, EU‐27 public opinion might become even more relevant in this second phase of Brexit negotiations than during the withdrawal negotiations.

It is therefore important to understand EU‐27 public opinion on Brexit. This article contributes to this goal by providing insights on European voters' views on the Brexit negotiations and the consequences of Brexit. It relies on survey data from 39,000 EU‐27 respondents , which I collected in four survey waves run at six‐month intervals between the start of the negotiations in the summer of 2017 and December 2018. The article argues that Brexit confronts the EU‐27 with a number of difficult choices. Losing the close cooperative relations between the UK and the EU will be costly, not only for the UK, but also for the remaining member states. At the same time, making the UK better off outside the EU raises the risk that more countries may be encouraged to leave the EU. This creates an *accommodation dilemma* (Jurado *et al.,* 2018; Walter, 2020b) for those EU‐27 Europeans who are exposed to the fallout from an uncompromising Brexit arrangement, but who also care about the long‐term stability of the EU. After a brief overview of the survey design and some descriptive evidence, the article explores in detail who supports a more accommodating stance and who supports a less compromising stance in the Brexit negotiations. It finds that EU‐27 Europeans understand that Brexit confronts them with an accommodation dilemma between maintaining the benefits of close cooperation with the UK and the risks of encouraging further disintegrative tendencies elsewhere. The conclusion discusses what these insights on EU‐27 public opinion imply for the Brexit process.

## I Brexit Risks, the Accommodation Dilemma and EU‐27 Negotiation Preferences

Brexit marks a turning point in EU history. For the first time an EU member state has left the EU, leading to concern that Brexit may pose a serious threat to the EU as a whole (Laffan, 2019). After all, Brexit puts the integrity of the single market in jeopardy (Jensen and Kelstrup, 2019) and diminishes the EU's global standing (Bulmer and Quaglia, 2018). Moreover, Brexit carries significant spillover effects in the other EU member states. Two types of spillover effects are particularly important: first, the loss of cooperation gains that disintegration entails, and second, the risk of political contagion. Whether and to what extent these spillover effects will materialize, however, depend greatly on the outcome of the Brexit negotiations between the UK and the EU. As a result of this, EU‐27 Europeans' negotiation preferences will be informed by how exposed they are to these risks and by how they evaluate them.

### Spillover Effects: Costly Non‐Cooperation vs Political Contagion

Many of the gains from cooperation that are now endangered by Brexit are economic in nature, such as the potential damage to firms engaged in trade with the UK, or the economic downturn and job losses that are likely to occur if trade ties between the EU and the UK are cut or significantly reduced (Hix, 2018). Other costs of Brexit include, among other things, the loss of London's contributions to the EU budget, or the loss of free access to Europe's financial centre. However, many costs are also social or political in nature, such as travel restrictions between the UK and the EU‐27, the end of the free movement of people, uncertainty about the future of EU citizens living in the UK, or the exclusion of the UK from EU‐wide anti‐crime or anti‐terrorism schemes. If Brexit significantly severs the strong ties between the EU and the UK, it will thus impose considerable costs on the EU‐27 public.

Nonetheless, the level of these costs is likely to vary significantly among individuals and countries. They are highest for individuals who benefit from a close exchange with the UK, either directly in personal or business terms, or indirectly through their regional economy. For example, for individuals who live in member states that are closely integrated with the UK, the costs of Brexit are going to be larger than for those in countries whose ties with the UK are more limited. This exposure can vary considerably. A hard Brexit, for example, is estimated to put less than 0.5 per cent of Slovakia's and Bulgaria's GDP at risk, but more than 10 per cent of Irish and more than 5 per cent of German GDP (Chen *et al.,* 2018). 4 The potential spillover effects caused by the loss of cooperation gains were already considerable during the withdrawal negotiations, and will become a defining issue in the negotiations about the future EU‐UK relationship. But how exactly these spillover effects will play out will depend to a great deal on how the future relationship between the EU and the UK is ultimately designed.

A second spillover effect is political in nature. A successful Brexit that makes the UK better off outside the EU will demonstrate to citizens of the other EU member states that it is possible for countries to improve their position unilaterally, while still enjoying many of the benefits of EU membership (De Vries, 2017; Hobolt, 2016; Walter, 2020b). Research has shown that individuals tend to benchmark their own government's performance (Kayser and Peress, 2012) and the desirability of EU membership (De Vries, 2018) across borders, that is, they take other countries' experiences into account in their assessments. By providing a powerful counterfactual that demonstrates that voters abroad no longer support European integration and that allows people to benchmark accurately the extent to which disintegration presents their country with a viable and better alternative to membership in the EU, a successful Brexit is likely to encourage disintegrative tendencies in other member states (De Vries, 2017; Malet, 2019). This could come in the form of support for further withdrawals from the EU, but also in the form of increased requests for country‐specific EU rules, which could, over time, undermine the EU's cohesiveness.

At the same time, however, observing that the UK is worse off post‐disintegration is likely to deter voters from seeking an exit of their own country. By providing a reality check, Brexit thus also has the potential to make an EU exit less attractive, especially for voters who expect that leaving the EU would allow them to enjoy both the benefits of international cooperation and regained national sovereignty at the same time. 5 Although developments since the 2016 Brexit referendum suggest that the effect of Brexit so far has been more of a deterrence than an encouragement on the EU‐27 public (Glencross, 2019), this discussion implies that the ultimate effects of Brexit on political contagion dynamics will depend in no small part on how the UK fares post‐Brexit (Walter, 2020a, 2020b).

### An Accommodation Dilemma for the EU‐27

The degree to which these two types of spillover effects will manifest themselves depends on the way the UK's withdrawal process is handled and on the contours of the future relationship between the EU and the UK. This confronts the EU‐27 side with a dilemma. On the one hand, cooperation losses will be smaller the closer the relations between the two remain, creating incentives for the EU to salvage as many of the cooperation gains from the existing arrangement as possible by accommodating many of the UK's requests. This could mean, for example, granting the UK significant access to the single market while allowing the UK to restrict the free movement of people or to deviate from level playing field provisions, or allowing it to continue participating in common programmes such as those on police or research cooperation. 6

On the other hand, the extent and direction of political contagion effects – encouragement or deterrence – will depend on how attractive the UK's new model will be for other member states. An outcome that accommodates many British requests and therefore allows the UK to enjoy many of the benefits from EU integration without major strings attached, may minimize the economic costs of Brexit. However, it risks undermining the long‐term stability of the EU, both in terms of the integrity of the single market, but also in terms of possible further member state withdrawals. A non‐accommodative stance that is uncompromising and makes exit costly for the UK, in contrast, is likely to deter disintegrative tendencies. 7

As a result, the EU institutions, the EU‐27 governments and large parts of the EU‐27 public face an accommodation dilemma (Jurado *et al.,* 2018; Walter, 2020b). On the one hand, a hard, non‐accommodating negotiation outcome – or even a no‐deal scenario – would be costly for the remaining member states, even if the costs are lower in scale than for the UK. But, at the same time, making the UK better off outside the EU by allowing it to enjoy the benefits of EU integration without sharing the costs threatens the long‐term stability of the EU.

### EU‐27 Negotiation Preferences

I argue that the way individuals, in the face of the accommodation dilemma, view the Brexit negotiations, and whether they support a more accommodating or a more hard line negotiation approach by the EU depends on how exposed they are to the consequences of each of the two types of spillover effects. Overall, individuals should be particularly hawkish when they can expect the net costs of non‐accommodation to be small for them, but more dovish when the costs of non‐accommodation outweigh the benefits of taking a hard negotiating line. This means that individuals who are more exposed to the losses of cooperation gains from a hard Brexit – be it because they have personal ties to the UK or because they live in an economy that is particularly vulnerable to a hard Brexit – should be more supportive of a softer, more accommodating approach. In contrast, those with little exposure should take a tougher stance.

At the same time, those who are most concerned about preserving the long‐term stability of the EU should support a more hawkish negotiating stance. The more positively individuals view the EU, the less willing they should be to accommodate the UK. In contrast, creating an attractive EU‐exit blueprint should appeal to eurosceptics, especially if they aspire to a withdrawal of their own country from the EU. I therefore expect more eurosceptic individuals to support a more accommodative stance towards the UK.

The accommodation dilemma should moderate these relationships. Europhile Europeans concerned about political contagion risks should be particularly uncompromising when their exposure to the fallout from a hard Brexit is low, but should be more accommodating when it is high. Eurosceptics, in contrast, face no dilemma: I expect them to support a more accommodating stance across the board.

## II EU‐27 Public Opinion on Brexit: Research Design and Descriptive Evidence

To analyse Brexit‐related public opinion in the EU‐27, I use survey data from about 39,000 EU‐27 respondents of working age collected in four survey waves run in six‐month intervals between the start of the negotiations in the summer of 2017 and December 2018. 8 The data were collected by placing questions on an EU‐wide online survey omnibus (the EuroPulse), regularly conducted by Dalia Research. 9 In each wave, the sample consists of a census representative sample of between 9,000–10,000 working‐age respondents (aged 18–65 years). Respondents were drawn across the remaining 27 EU member states, with sample sizes roughly proportional to their population size. 10 In order to obtain census representative results, the data are weighted based upon the most recent Eurostat statistics. 11 For the detailed regression analyses I rely on the most recent survey wave from December 2018 because it contains information about respondents' location. For these analyses I use hierarchical three‐level models that take account of the nested structure of the data (individuals nested in regions nested in countries). 12

### Negotiating Brexit: Europeans' Negotiation Preferences

To gauge individuals' preferences on the EU's Brexit negotiation strategy, that is, whether respondents support an accommodating, softer EU negotiation stance in the Brexit negotiations or a harder, non‐accommodating approach, I asked respondents directly how they thought the EU should approach the exit negotiations with the UK. 13 The question defined a hard (non‐accommodating) line in the Brexit negotiations as one in which the EU insists that the UK pay a large "exit bill" to compensate the EU for the costs of Brexit, guarantees special rights for EU citizens living in the UK, and ensures that the UK does not get privileged access to the European single market. In contrast, it defined a soft (accommodating) line as a negotiation position that accepts that the UK pays only a small exit bill, allows the UK to limit the rights of EU citizens currently living in the UK, and gives the UK privileged access to the European single market. Respondents were asked to report their preferred negotiation line on a five‐point scale ranging from (1) ‘very soft line’, to (5) ‘very hard line’. 14

**Figure 1:**
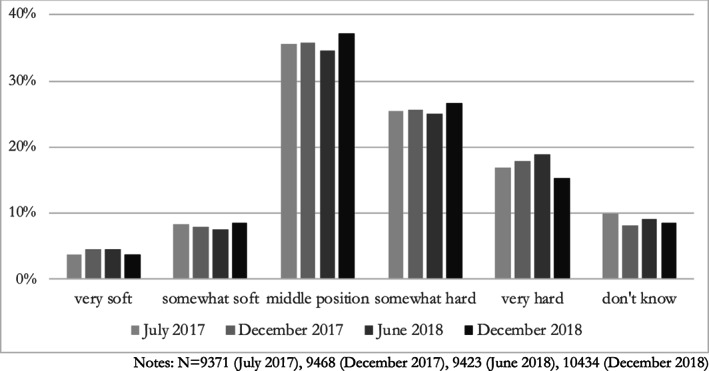
Preferred EU Negotiation Stance, July 2017–December 2018

Figure 1 presents respondents' Brexit negotiation preferences over the first two years of the Brexit withdrawal negotiations. It shows that support for a (very) soft, accommodating EU negotiation strategy was always low. A good third of respondents would prefer the EU to take a middle position between a soft and a hard line, and this group grew slightly over the course of the Brexit negotiations. Nonetheless, from the start of the negotiations, Europeans have on average preferred a hard, non‐accommodating Brexit negotiation strategy. Between 42 and 44 per cent of respondents supported a hard or very hard negotiation stance in each of the four survey waves. Only when the difficulties of successfully concluding the withdrawal agreement increased in December 2018 did respondents slightly move towards a more compromising stance.

Overall, the descriptive evidence shows that – contrary to statements by some UK Brexiteers that ‘lots of Europeans are uneasy at the line the EU Commission is taking on Brexit’ 15 – the EU's uncompromising negotiation strategy was supported by many European citizens. In the analyses below, I use the question about the preferred soft or hard negotiation line as dependent variable, with higher values indicating a preference for a harder, non‐accommodating negotiation strategy.

### Correlates of Preferring a Hard Negotiation Line

I argue that variations in willingness to accommodate the UK in Brexit negotiations is related to how individual EU‐27 Europeans are exposed to the economic and political spillover effects associated with different Brexit negotiation outcomes and how they evaluate these effects. To examine the correlates of individuals' support for a hard, non‐accommodating Brexit negotiation strategy on part of the EU, I operationalize exposure to the loss of cooperation gains and the concern about Brexit‐related contagion risks as follows.

#### Exposure to Loss of Cooperation Gains

**Figure 2:**
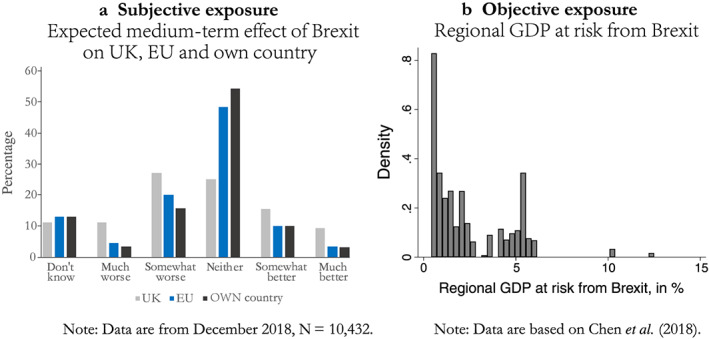
Distribution of Respondents' Exposure to the Consequences of Brexit [Colour figure can be viewed at wileyonlinelibrary.com]

To measure individuals' exposure to Brexit‐related losses of cooperation gains, I focus both on subjective and objective exposure. Respondent's subjectively perceived exposure to Brexitis measured with their assessment about how Brexit will affect their own country within five years on a five‐point scale, where higher values indicate that respondents anticipate more negative effects on their own country. 16 Figure 2a shows how this variable is distributed and compares respondents' assessment of the effects of Brexit on their own country to those on the UK and the EU. It demonstrates that as late as December 2018, the majority of respondents were somewhat unconcerned about the effects of Brexit on their own country. 17 More than half (54.6%) did not think that Brexit would affect their own country at all, and 13.3 per cent even thought that Brexit would make their country (much) better off. Only 19.2 per cent thought that their own country would be somewhat or much worse off because of Brexit. In contrast, 48 per cent expected that Brexit would affect the UK negatively. That said, a quarter of respondents also expected that the UK would be better off post‐Brexit, and about a quarter did not expect any effect at all. Respondents were more optimistic about the effects of Brexit on the EU, although on average they believed that the EU faces slightly more risks than their own country. 18

Given this rather optimistic assessment, I additionally use an objective indicator of the risks that Brexit poses to respondents' regional economy. Chen *et al*. (2^2018^: Table A2) have estimated the degree to which EU regions are exposed to the negative trade‐related consequences of Brexit that arise from the geographically fragmented production processes within the UK, the EU and beyond. I use their estimates of the regional GDP at risk from (a hard) Brexit and match it to the survey data using information about the respondent's location, matching regions on level 2 of the nomenclature of territorial units for statistics (NUTS). 19 Figure 2b shows the distribution of objective Brexit exposure among the respondents in my sample. Regional exposure to Brexit‐related trade losses varies from only 0.41 per cent of regional GDP at risk in Liguria (Italy) to 10.13 per cent in the Irish border region, Irish midlands, and western Ireland. 20 The median exposure of EU‐27 respondents in my sample is 1.5 per cent of regional GDP at risk. As the data are highly skewed, I use the logarithm of this variable in the analyses below.

Finally, I look at respondents' objective direct exposure, using a dummy variable that records whether respondents had personal or business ties (including through their employer) with the UK. While four in five respondents report no ties, 11.5 per cent report personal ties, 4.5 per cent report business ties, and 4 per cent report both personal and business ties.

#### Concern about Contagion Risks

A second type of spillover effect from Brexit consists in the possibility that Brexit may spark off political contagion. This is a worrisome prospect for those who value the EU and want to safeguard the European integration project. I therefore expect such individuals to support a harder, non‐accommodating negotiation stance. For eurosceptics, however, an outcome that allows the UK to continue to selectively benefit from the advantages of EU membership post‐Brexit is attractive, especially if they see an exit from the EU as a desirable outcome for their own country. They should thus be more willing to accommodate the UK.

I use two variables to capture these considerations. First, at the most basic level, I look at respondents' overall attitude towards the EU, using the question ‘What is your opinion of the EU?’ Answers on the five‐point scale ranged from 0 ‘very negative’ to 4 ‘very positive’. 21 Second, I look at how respondents said they would vote if a referendum on leaving the EU were to be held in their own country. I create a dummy variable that takes the value of 1 if respondents said that they would definitely (10.6%) or probably (13.9%) vote to leave the EU, and 0 otherwise.

#### Other Controls

I also control for respondents' level of information, political participation and sociodemographics. Given the multidimensional and complicated nature of Brexit, one would expect the better informed respondents to understand better the many dilemmas and trade‐offs it creates. It is not clear a priori, however, whether this will result in a more or less accommodating stance towards the UK. On the one hand, more information about the difficulties in finding a compromise and the risks of a negotiation failure may increase respondents' willingness to accommodate the UK. On the other hand, more information about the political contagion risks of Brexit for the EU may also lead to a harder stance. I use a variable that measures how much respondents follow the news about Brexit. Only 17.7 per cent follow it a lot, but 49.9 per cent follow it at least a little. About one quarter does not pay it a lot of attention, and 8.2 per cent say that they do not follow Brexit‐related news at all.

Politicians tend to pay more attention to potential voters, whereas the interests of non‐voters are more readily dismissed (Walter, 2016). For the Brexit negotiations, this means that the opinions of those EU‐27 citizens who are likely to turn out and vote are likely to carry more political weight than the preferences of the politically uninterested public. To gauge whether the preferences over Brexit negotiations of more politically active respondents are different from those of less politically active respondents, I include a dummy variable that takes the value of 1 if an individual reports that they are certain to vote in the next national election. Finally, I control for sociodemographic variables: age, gender, education, and whether the respondent lives in a rural or urban setting.

## III Why EU‐27 Europeans' Willingness to Accommodate the UK Varies

**Figure 3:**
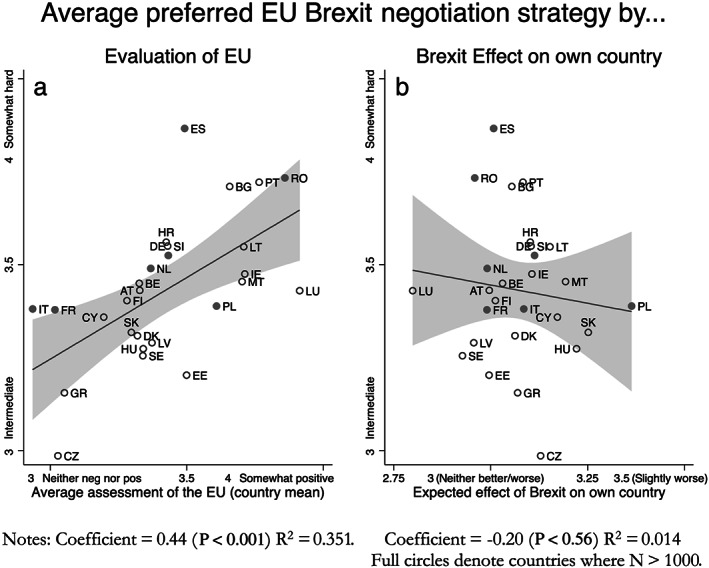
Country‐level Variation in Preferences for a Hard EU Brexit Negotiation Strategy

Why are some EU‐27 Europeans more willing to accommodate the UK than others? Figure 3 pools all waves and plots the average preferred EU‐Brexit negotiation strategy for each EU‐27 member state relative to the average evaluation of the EU (left‐hand panel) and the average subjective assessment about the medium‐term consequences of Brexit for respondents' own country (right‐hand panel). The figure documents significant country‐level variations in EU‐27 Brexit negotiation preferences. Figure 3a shows that, as suggested by the accommodation dilemma, countries in which respondents view the EU more positively on average support a more uncompromising approach in Brexit negotiations. In contrast, a country's vulnerability to the consequences of Brexit is not strongly related to negotiation preferences (see Figure 3b).

To evaluate these relationships more systematically, Table 1 shows the results from a regression analysis of how respondents' exposure to the spillover effects of Brexit are related to their Brexit negotiation preferences, using data from the December 2018 wave. The key finding from the analysis is that, as expected, both exposure to the loss of cooperation gains and concern about the stability of the EU are associated with respondents' preferred EU stance in the Brexit negotiations. Columns 1 and 2 show two unconditional models. These analyses find that those who are more exposed to the negative consequences of Brexit, both in subjective and objective terms, are significantly more accommodating towards the UK than those who are less exposed. The one exception are those with personal ties to the UK, who support a significantly harder line, possibly because a hard line was defined as including better protection for the rights of EU citizens in the UK. Somewhat surprisingly, business ties have no statistically significant effect. 22

**Table 1:** Correlates of Brexit Negotiation Preferences (Three‐level Multilevel Models)

	Model 1	Model 2	Model 3	Model 4
**Exposure to loss of cooperation gains**				
Expected Brexit effect on own country	−0.155***P 0 (0.03)		−0.081 (0.07)	
Regional GDP at risk from Brexit (logged)		−0.097* (0.05)		0.130 (0.09)
Personal ties to UK	0.080** (0.04)	0.100** (0.04)	0.075** (0.04)	0.099** (0.05)
Business ties to UK	0.045 (0.06)	0.090 (0.06)	0.040 (0.06)	0.096 (0.06)
**Assessment of political contagion**				
General opinion of EU	0.194***P 0 (0.04)	0.253***P 0 (0.04)	0.295***P 0 (0.10)	0.294***P 0 (0.02)
Potential Leave voter	−0.174***P 0 (0.05)	−0.050 (0.04)	−0.176***P 0 (0.05)	−0.053 (0.04)
**Interaction effects**				
Expected Brexit effect * EU opinion			−0.032 (0.02)	
Regional GDP at risk * EU opinion				−0.093***P 0 (0.02)
**Controls**				
Attention to Brexit news	0.255***P 0 (0.02)	0.360***P 0 (0.02)	0.254***P 0 (0.02)	0.361***P 0 (0.02)
Certain to vote in next election	0.174***P 0 (0.04)	0.231***P 0 (0.03)	0.174***P 0 (0.04)	0.231***P 0 (0.04)
Age	−0.002 (0.00)	−0.004* (0.00)	−0.002 (0.00)	−0.004* (0.00)
Education	0.050***P 0 (0.02)	0.082***P 0 (0.03)	0.050***P 0 (0.02)	0.083***P 0 (0.03)
Female	−0.096* (0.05)	−0.160***P 0 (0.04)	−0.095* (0.05)	−0.163***P 0 (0.05)
Rural	0.059 (0.04)	0.045 (0.04)	0.060 (0.04)	0.045 (0.04)
Constant	2.471***P 0 (0.18)	1.430***P 0 (0.17)	2.239***P 0 (0.31)	1.328***P 0 (0.14)
**Random effects**				
Country‐level variance	0.022 (0.011)	0.015 (0.006)	0.021 (0.011)	0.013 (0.006)
Region‐level variance	0.022 (0.008)	0.023 (0.010)	0.022 (0.008)	0.023 (0.010)
Log likelihood	−13498.9	−16167.5	−13495.3	−16140.662
N (countries)	27	26	27	26
N (regions)	244	243	244	243
N (individuals)	9,006	10,103	9,006	10,103

*Notes*: Dependent variable is thefive‐point measure of preferred EU Brexit negotiation line, with higher values denoting a preference for a harder, less accommodating stance. Multilevel model using weighted data. Standard errors in parentheses.

^*^ *P* < 0.1

^**^ *P* < 0.05

^***^ *P* < .001

While concern about the costs of a hard Brexit softens EU‐27 Europeans' preferred negotiating stance, the possibility of political contagion effects also matters. The more positively they view the EU, the harder and less accommodating their stance towards the UK becomes. At the same time, those who themselves favour an exit of their own country from the EU are much more accommodating towards the UK. This is not surprising, because Brexit offers an opportunity to establish a precedent that is favourable towards the withdrawing state. 23

I next examine to which extent the accommodation dilemma shapes EU‐27 Europeans' Brexit negotiation preferences. This dilemma confronts Europeans who worry that accommodating the UK may encourage further exits from the EU, but who at the same time are vulnerable to economic or social fallout from a hard Brexit. To explore the extent to which a greater exposure to the economic and social fallout from Brexit moderates EU‐27 Europeans' concern about political contagion effects, and vice versa, models 3 and 4 include interaction terms between the sociotropic exposure variables and respondents' assessment of the EU.

**Figure 4:**
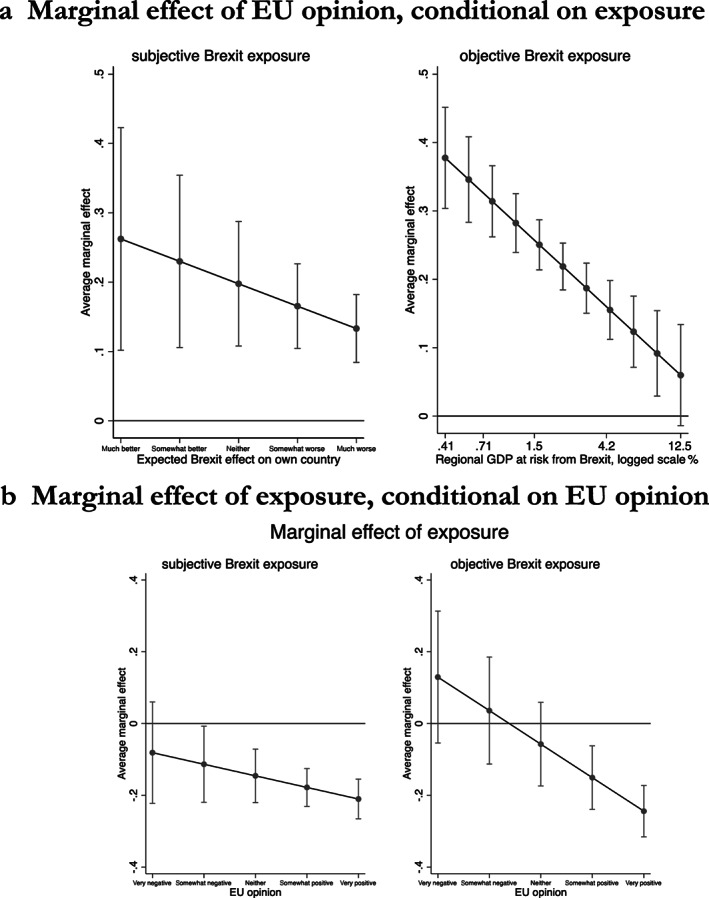
a: Marginal Effect of EU Opinion, Conditional on Exposure, b: Marginal Effect of Exposure, Conditional on EU Opinion

The negative interaction terms show that EU‐27 Europeans do indeed experience an accommodation dilemma. A more positive view of the EU makes respondents significantly less willing to accommodate the UK; yet exposure to the risks of Brexit moderates this effect. The interaction term is statistically significant at the 1 per cent level for the objective exposure measure, and barely misses statistical significance for the subjective measure (*P* < 0.102). To facilitate the interpretation of the interaction term, Figure 4a illustrates the effects of holding a more positive opinion of the EU, conditional on exposure to the perceived (left‐hand panel) and the objective (right‐hand panel) exposure of a respondents' economic environment. It shows that, as expected, Europhile respondents are particularly hawkish when their exposure to the costs of non‐accommodating the UK is small. However, they become more dovish when their exposure to the costs of non‐accommodation rises. This implies that those who face less of an accommodation dilemma (because they are Europhile but not exposed) are freer to concentrate on the political spillover effects of Brexit. In contrast, respondents for whom Brexit has potentially significant consequences need to confront the accommodation dilemma much more directly and therefore exhibit more moderate negotiation preferences. Moreover, Figure 4b shows that exposure moderates negotiation preferences only among Europhiles. As expected, only Europhiles experience an accommodation dilemma, whereas eurosceptics support accommodation irrespective of their exposure.

Finally, the analyses presented in Table 1 reveal another noteworthy finding. Respondents who are politically active take a particularly hard stance towards the UK. Those who pay more attention to the Brexit process take a significantly more uncompromising stance towards the UK than those who are less well informed. Likewise, those who plan to vote in the next national election support a harder negotiation line than those who are not sure they will vote in the next election. This is potentially bad news for the UK, because it suggests that EU‐27 citizens who are more politically influential are even less willing to accommodate the UK's requests than the average EU‐27 citizen. Finally, better educated respondents are less willing and women are more willing to accommodate the UK in Brexit negotiations.

## IV What Do EU‐27 Europeans Want from the Brexit Negotiations?

So far, we have seen that, on average, EU‐27 Europeans support the relatively hard negotiation line pursued by the EU in the Brexit negotiations. To understand what EU‐27 Europeans hope to achieve, I next examine their goals for the Brexit negotiations. In the December 2018 survey wave I asked respondents to rank five possible goals for the Brexit negotiations. Table 2 lists how often each of these goals was ranked as the most important goal. The first column shows the overall distribution of the answers, whereas the last two columns show how Europhiles and Eurosceptics, respectively, rank these goals.

**Table 2:** Respondents’ Ranking of Each Goal as the Most Important Brexit Negotiation Goal (%)

	All	Europhiles	Eurosceptics
Maintain my country's trade relations with the UK	34.9	26.6	30.0
Avoid that other countries leave the EU in the future	24.2	39.3	4.5
Establish a standard procedure that makes it easier for countries to leave the EU in the future	19.1	8.9	50.9
Avoid a failure of the Brexit negotiations	14.8	14.2	9.9
Punish the UK for leaving the EU	7.0	11.0	4.7
N	10,432	1,166	815

*Notes*: Europhiles and eurosceptics are operationalized as those who see the EU as very positive or very negative, respectively.

Data are from the December 2018 survey.

The results shown in Table 2 confirm that overall, the EU‐27 public is indeed concerned about the economic and political spillover effects of Brexit on the EU and their own countries. The goal that respondents most frequently ranked as most important was to ‘maintain their country's trade relations with the UK’. For one in three respondents, limiting the economic fallout from Brexit is thus the core objective of the Brexit negotiations. The two runners‐up focus on political spillovers: avoiding and encouraging political contagion were the second and third most frequent top goals for the Brexit negotiations. Every fourth respondent said that the most important objective was to avoid encouraging other countries to follow the British example, whereas one in five stated that it was most important to make it easier for countries to leave the EU in the future. Only seven per cent of respondents listed punishing the UK for the decision to leave the UK as the most important goal.

However, Table 2 also shows that there is considerable variation in what Europhiles and eurosceptics want to achieve in the Brexit negotiations. For Europhiles, avoiding that other countries follow the UK's example was the most important goal. In contrast, for a majority of eurosceptics (50.1%) 24 establishing a blueprint that would make leaving the EU easier in the future was the most important goal. This suggests that fears about the risk of political contagion are not unfounded. Although it has been argued that the contagion risks of Brexit have subsided since the Brexit referendum (Chopin and Lequesne, 2020; Glencross, 2019), as late as December 2018 eurosceptic voters were acutely aware that Brexit offers an opportunity for a precedent that could facilitate exiting in the EU in the future. This also means that a favourable long‐term Brexit outcome for the UK might indeed encourage eurosceptics in the remaining EU‐27 member states to pursue EU‐exit plans themselves.

## Conclusion

In the Brexit withdrawal negotiations, British hopes that the remaining EU countries were willing to offer the UK better withdrawal terms than the EU Commission have been repeatedly frustrated. Instead, the EU‐27 governments have been united in rejecting any British attempts at cherry‐picking, even at the risk that their uncompromising stance might result in a no‐deal Brexit. This article has shown that the EU‐27 has good reasons to maintain this tough negotiation stance. Not only does the EU‐27 side have more bargaining power because the UK is more vulnerable to a failure to reach a deal (Moravcsik, 2018; Schimmelfennig, 2018). The EU's tough line can also be explained by the concern that making it possible for the UK to enjoy the benefits of EU integration without sharing the costs might encourage support for EU withdrawal among further member states. Because accommodating the UK carries significant risks of political contagion, the EU thus has incentives to make the exit of a member state as unattractive as possible. Against this background, the tough line taken by the EU side comes as less of a surprise.

This article has shown that support for the EU's uncompromising negotiation stance in the withdrawal negotiations has not been limited to political elites. Rather, it has been supported by the wider EU‐27 public. Using evidence from several EU‐wide online surveys of EU‐27 Europeans fielded during these negotiations, I have shown that the EU‐27 public on average supported a somewhat hard negotiation stance. Their most important goal was to maintain their respective country's trade ties with the UK, but they also worried that allowing the UK to cherry‐pick its most‐liked aspects of EU membership would threaten the long‐term stability of the EU. At the same time, eurosceptics are indeed eager to use the Brexit negotiations to develop a blueprint that makes it easier for countries to leave the EU in the future. Importantly, support for a hard negotiation stance is stronger among respondents who are more likely to turn out to vote. Policy‐makers responsive to public opinion thus have incentives to continue to pursue a non‐accommodating negotiation line.

Moreover, the analyses in this article show that the EU‐27 public seems to recognize the trade‐offs inherent in the Brexit negotiations and have formed their preferences on the negotiations accordingly. The more exposed individuals are to the potential fallout from Brexit, the more likely they are to compromise. The more they care about the viability of the EU, the less accommodating they are. These goals often also conflict, and the evidence shows that the accommodation dilemma moderates Europeans' Brexit‐related preferences. Overall, there is evidence of an EU‐27 public that is well aware of the consequences of Brexit, and rather unsentimentally supports a negotiation line that safeguards its own interests best.

More generally, the evidence shows just how difficult ‘voter‐endorsed disintegration’ (Walter, 2020b) is. Recent successes by nationalist populists at the polls – such as the 2014 Swiss ‘against mass immigration’ initiative, the 2015 Greek bailout referendum, or the 2016 election of US President Trump – have often been based on a common narrative that by being more assertive in international relations and putting the nation's interest first rather than accepting compromise, the country's prosperity, national sovereignty, and democratic quality could be improved. This narrative has usually not survived the test of reality, however, as successes in domestic polls have been met with resistance abroad. Renegotiating international agreements has proven difficult, if not impossible, and has sometimes forced populist governments to concede that the status quo is better than what they could achieve if they left the agreement. Ultimately, voter‐endorsed attempts to unilaterally change or withdraw from the rules of international cooperation have not failed because of poor negotiation skills on part of the governments of the withdrawing states, but because voters and elites in other countries have been unwilling to grant special privileges to another state at their own expense.

## Supporting information


**Figure S1:** Comparison of share of respondents viewing the EU somewhat/very positively between Dalia Sample from December 2018 wave and Eurobarometer November 2018 wave.
**Figure S2a:** Robustness check for Figure 4a to using OLS, country fixed effects, SEs clustered at country level (figure based on model 3, table S3).
**Figure S2b:** Robustness check for Figure 4b to using OLS, country fixed effects, SEs clustered at country level (figure based on model 3, table S3).
**Figure S3a:** Robustness check for Figure 4a to using OLS, country fixed effects, SEs clustered at regional level (figure based on model 3, table S4).
**Figure S3b:** Robustness check for Figure 4b to using OLS, country fixed effects, SEs clustered at regional level (figure based on model 3, table S4).
**Table S1:** Correlation coefficients between Dalia and Eurobarometer data.
**Table S2:** Correlations.
**Table S3:** Robustness check for Table 1, using OLS, country fixed effects, SEs clustered at country level.
**Table S4:** Robustness check Table 1, using OLS, country fixed effects, SEs clustered at regional level.

## Footnotes

^1^

The public also penalizes policy‐makers for backing down in international negotiations and confrontations (e.g., Tomz, 2007).

^2^

Moreover, parliamentarians are equally likely to respond to the preferences and views of their voters.

^3^

The exact ratification requirements will depend on the nature of the agreement. National parliaments will have to ratify any agreement that refers to competences that the EU shares with member states.

^4^

For other estimates of the Brexit‐related fallout in the EU‐27 see Lawless and Morgenroth (2019).

^5^

This belief is relatively widespread. See Milic (2015); Owen and Walter (2017); Sciarini *et al*. (2015); Walter *et al*. (2018).

^6^

These exceptions are likely be costly to the EU as well, because they would change the distribution of cooperation gains between the UK and the EU. Nonetheless, they are still likely to be less costly in economic terms than a breakdown of the negotiations and a resulting No‐Deal‐Brexit.

^7^

In the negotiations, such a strategy moreover serves to signal resolve and can be used to convince the other side to soften its demands.

^8^

The surveys thus cover the negotiations about the withdrawal agreement, not the future relationship.

^9^

This omnibus has been used by other researchers (see De Vries, 2017, 2018, 2019; Karstens, 2019). De Vries (2018, p. 66, footnote 6) notes that the demographic background of EuroPulse survey respondents shows very little difference from nationally representative surveys. Additional analysis (see online appendix, Figure S1 and Table S1) show that average country‐level EU support in the November 2018 Eurobarometer and the December 2018 EuroPulse survey are correlated, especially for countries with a sample size larger than 300. While the EuroPulse contains data for the UK, these are omitted in my analyses.

^10^

Table A1 in the appendix reports the sample sizes by country.

^11^

The target weighting variables are age, gender, level of education (as defined by ISCED 2011 (UNESCO Institute for Statistics, 2012)) levels 0–2, 3–4, and 5–8), and degree of urbanization (rural and urban).

^12^

Intraclass correlations are 2.5 per cent at the country level, and 4.0 per cent at the region level. Results are robust to using an ordinary least squares regression model with clustered standard errors clustered at the country level (see Table S3 and Figures 2a+b in the supplementary materials for SEs clustered at the country level and Table S4 and Figures 3a+b ifor SEs clustered at the regional level).

^13^

This question immediately followed upon an introductory question that asked how much attention respondents were paying to Brexit.

^14^

The category ‘Don't know/don't answer’ was recoded as missing for most analyses. The issues covered in the question are deliberately broad, covering both issues from the withdrawal and the future relationship negotiations.

^15^

See for example https://twitter.com/DanielJHannan/status/1046677612939137024

^16^

The effect of Brexit will, of course, depend on the type of Brexit negotiated, so that answers to this question will vary depending on which outcome respondents envision for the Brexit process.

^17^

The earlier surveys show a similar picture, with very little movement over the two years of Brexit negotiations.

^18^

Note that, although those who were more interested in Brexit expected significantly worse consequences for the UK than those who did not follow the news on Brexit much, they shared the low level of concern on the consequences of Brexit for the UK and the EU.

^19^

Unfortunately, the authors do not provide any estimates for Croatia, which is why this country was dropped from the analyses that use this objective measure of exposure.

^20^

The results are robust to using regional labour income at risk instead (Chen *et al.,* 2018: Table A4).

^21^

Altogether 24.6 per cent of respondents had somewhat or very negative opinion, 27.2 per cent neither a positive nor negative, and 48.2 per cent had a somewhat or very positive opinion.

^22^

Note that the effect becomes statistically significant when personal ties are excluded. This is not surprising, given that personal and business ties are strongly correlated in my sample (see Table S2, online appendix).

^23^

This effect loses statistical significance in the models controlling for objective exposure, but retains statistical significance if the variable on EU opinion, with which the leave dummy is strongly correlated, is omitted.

^24^

Among those who say that they would probably vote to leave, this share is 34.4 per cent.

## Appendix

**Table A1:** Sample composition by country

Country	December 2018 wave	All waves
Austria	200	761
Belgium	239	948
Bulgaria	149	618
Cyprus	20	83
Czech Republic	286	950
Germany	1784	6,585
Denmark	114	489
Estonia	27	113
Spain	1,072	4,276
Finland	108	453
France	1,311	5,190
Greece	223	921
Croatia	91	350
Hungary	198	827
Ireland	102	391
Italy	1,242	4,973
Lithuania	55	243
Luxembourg	17	58
Latvia	40	159
Malta	10	45
Netherlands^*^	1,035	2061
Poland	1,087	4,200
Portugal	237	883
Romania	408	1,628
Sweden	203	806
Slovenia	48	184
Slovakia	128	501
Total	10,434	38,696

^*^ In December 2018, Dalia Research increased the Dutch sample size to over 1,000. The weights are adjusted accordingly.
