# Supplementary material for: EU‐27 Public Opinion on Brexit
Source: J Common Mark Stud. 2020 Dec 4;59(3):569–88. doi: 10.1111/jcms.13107 (PMC9292232; doi:10.1111/jcms.13107)
Supplement: Supplementary file 1 — Figure S1: Comparison of share of respondents viewing the EU somewhat/very positively between Dalia Sample from December 2018 wave and Eurobarometer November 2018 wave. Figure S2a: Robustness check for Figure 4a to using OLS, country fixed effects, SEs clustered at country level (figure based on model 3, table S3). Figure S2b: Robustness check for Figure 4b to using OLS, country fixed effects, SEs clustered at country level (figure based on model 3, table S3). Figure S3a: Robustness check for Figure 4a to using OLS, country fixed effects, SEs clustered at regional level (figure based on model 3, table S4). Figure S3b: Robustness check for Figure 4b to using OLS, country fixed effects, SEs clustered at regional level (figure based on model 3, table S4). Table S1: Correlation coefficients between Dalia and Eurobarometer data. Table S2: Correlations. Table S3: Robustness check for Table 1, using OLS, country fixed effects, SEs clustered at country level. Table S4: Robustness check Table 1, using OLS, country fixed effects, SEs clustered at regional level. [file JCMS-59-569-s001.docx]

**EU-27 Public Opinion about Brexit**

**Supplementary materials**

**(online appendix)**

**S1: Comparison between Dalia and Eurobarometer Samples**

**Figure S1: Comparison of share of respondents viewing the EU somewhat/very positively between Dalia Sample from December 2018 wave and Eurobarometer November 2018 wave.**

Table S1: Correlation coefficients between Dalia and Eurobarometer data

|  | **Pearson’s Correlation coefficient** | **N** |
| --- | --- | --- |
| Full sample | 0.4175 | 27 |
| Sample excluding countries with N<100 | 0.4941 | 19 |
| Sample excluding countries with N<300 | 0.9091 | 7 |

**S2: Correlations**

|  | preferred EU Brexit strategy | Expected Brexit effect | Regional GDP at risk (logged) | Personal ties | Business ties | EU opinion | Leaver | Brexit awareness | Certain to vote | age | education | female |
| --- | --- | --- | --- | --- | --- | --- | --- | --- | --- | --- | --- | --- |
| preferred EU Brexit strategy | 1 |  |  |  |  |  |  |  |  |  |  |  |
| Expected Brexit effect | -0.12 | 1.00 |  |  |  |  |  |  |  |  |  |  |
| Regional GDP at risk (logged) | -0.07 | 0.00 | 1.00 |  |  |  |  |  |  |  |  |  |
| Personal ties | 0.07 | -0.06 | 0.00 | 1.00 |  |  |  |  |  |  |  |  |
| Business ties | 0.05 | -0.07 | 0.04 | 0.25 | 1.00 |  |  |  |  |  |  |  |
| EU opinion | 0.28 | -0.13 | -0.01 | 0.10 | 0.07 | 1.00 |  |  |  |  |  |  |
| Leaver | -0.20 | 0.05 | 0.04 | -0.03 | 0.02 | -0.60 | 1.00 |  |  |  |  |  |
| Brexit awareness | 0.23 | -0.03 | -0.06 | 0.14 | 0.10 | 0.15 | -0.05 | 1.00 |  |  |  |  |
| Certain to vote | 0.13 | 0.02 | -0.07 | -0.03 | -0.06 | 0.08 | -0.05 | 0.26 | 1.00 |  |  |  |
| age | 0.01 | 0.01 | -0.06 | -0.14 | -0.14 | -0.10 | 0.06 | 0.13 | 0.18 | 1.00 |  |  |
| education | 0.09 | 0.02 | -0.21 | 0.09 | 0.04 | 0.11 | -0.10 | 0.17 | 0.13 | 0.03 | 1.00 |  |
| female | -0.06 | 0.06 | 0.01 | -0.02 | -0.08 | -0.01 | -0.02 | -0.10 | -0.02 | -0.01 | 0.05 | 1.00 |
| rural | -0.02 | 0.01 | 0.17 | -0.04 | 0.00 | -0.05 | 0.03 | -0.06 | -0.01 | 0.03 | -0.14 | 0.02 |

**S3a: Robustness Table 1, using OLS, country fixed effects, SEs clustered at country level**

|  | Model 1 | Model 2 | Model 3 | Model 4 |
| --- | --- | --- | --- | --- |
| **Exposure to loss of cooperation gains** |  |  |  |  |
| Expected Brexit-effect on own country | -0.153*** |  | -0.069 |  |
|  | (0.03) |  | (0.06) |  |
| Regional GDP at risk from Brexit |  | -0.419 |  | -0.195 |
| (logged) |  | (0.35) |  | (0.37) |
| Personal ties to UK | 0.080** | 0.098* | 0.056 | 0.097* |
|  | (0.04) | (0.05) | (0.04) | (0.05) |
| Business ties to UK | 0.037 | 0.081 | 0.068* | 0.087 |
|  | (0.06) | (0.06) | (0.04) | (0.06) |
| **Assessment of political contagion** |  |  |  |  |
| General opinion of EU | 0.191*** | 0.247*** | 0.283*** | 0.289*** |
|  | (0.04) | (0.04) | (0.07) | (0.03) |
| Potential Leave-voter | -0.185*** | -0.058 | -0.146*** | -0.061 |
|  | (0.05) | (0.04) | (0.05) | (0.04) |
| **Interaction effects** |  |  |  |  |
| Exp. Brexit-effect * EU opinion |  |  | -0.023 |  |
|  |  |  | (0.02) |  |
| Regional exposure * EU opinion |  |  |  | -0.094*** |
|  |  |  |  | (0.02) |
| **Controls** |  |  |  |  |
| Attention to Brexit news | 0.255*** | 0.357*** | 0.239*** | 0.359*** |
|  | (0.02) | (0.02) | (0.02) | (0.02) |
| Certain to vote in next election | 0.172*** | 0.233*** | 0.149*** | 0.233*** |
|  | (0.04) | (0.03) | (0.03) | (0.04) |
| Age | -0.002 | -0.004* | 0.000 | -0.004** |
|  | (0.00) | (0.00) | (0.00) | (0.00) |
| Education | 0.050*** | 0.083*** | 0.032* | 0.083*** |
|  | (0.02) | (0.03) | (0.02) | (0.03) |
| Female | -0.100* | -0.163*** | -0.100** | -0.166*** |
|  | (0.05) | (0.05) | (0.05) | (0.05) |
| Rural | 0.068 | 0.056 | 0.049 | 0.055 |
|  | (0.04) | (0.05) | (0.03) | (0.05) |
| Constant | 2.401*** | 1.275*** | 2.183*** | 1.175*** |
|  | (0.17) | (0.17) | (0.21) | (0.11) |
|  |  |  |  |  |
| R2 | 0.153 | 0.180 | 0.147 | 0.185 |
| Adjusted R2 | 0.149 | 0.177 | 0.144 | 0.182 |
| N (individuals) | 9006 | 10103 | 9006 | 10103 |

Note: country fixed effects not reported

**Figure S3a: Robustness Figure 4b, based on model S3**

**Figure S3b: Robustness Figure 4b, based on model S3**

**S4a: Robustness Table 1, using OLS, country fixed effects, SEs clustered at regional level**

|  | Model 1 | Model 2 | Model 3 | Model 4 |
| --- | --- | --- | --- | --- |
| **Exposure to loss of cooperation gains** |  |  |  |  |
| Expected Brexit-effect on own country | -0.153*** |  | -0.069 |  |
|  | (0.03) |  | (0.05) |  |
| Regional GDP at risk from Brexit |  | -0.419* |  | -0.195 |
| (logged) |  | (0.22) |  | (0.23) |
| Personal ties to UK | 0.080* | 0.098** | 0.056 | 0.097** |
|  | (0.04) | (0.04) | (0.03) | (0.04) |
| Business ties to UK | 0.037 | 0.081 | 0.068* | 0.087 |
|  | (0.05) | (0.05) | (0.04) | (0.05) |
| **Assessment of political contagion** |  |  |  |  |
| General opinion of EU | 0.191*** | 0.247*** | 0.283*** | 0.289*** |
|  | (0.02) | (0.02) | (0.05) | (0.02) |
| Potential Leave-voter | -0.185*** | -0.058 | -0.146*** | -0.061 |
|  | (0.05) | (0.05) | (0.04) | (0.05) |
| **Interaction effects** |  |  |  |  |
| Exp. Brexit-effect * EU opinion |  |  | -0.023 |  |
|  |  |  | (0.01) |  |
| Regional exposure * EU opinion |  |  |  | -0.094*** |
|  |  |  |  | (0.02) |
| **Controls** |  |  |  |  |
| Attention to Brexit news | 0.255*** | 0.357*** | 0.239*** | 0.359*** |
|  | (0.02) | (0.02) | (0.02) | (0.02) |
| Certain to vote in next election | 0.172*** | 0.233*** | 0.149*** | 0.233*** |
|  | (0.04) | (0.04) | (0.03) | (0.04) |
| Age | -0.002* | -0.004*** | 0.000 | -0.004*** |
|  | (0.00) | (0.00) | (0.00) | (0.00) |
| Education | 0.050** | 0.083*** | 0.032* | 0.083*** |
|  | (0.02) | (0.02) | (0.02) | (0.02) |
| Female | -0.100*** | -0.163*** | -0.100*** | -0.166*** |
|  | (0.03) | (0.04) | (0.03) | (0.04) |
| Rural | 0.068* | 0.056 | 0.049* | 0.055 |
|  | (0.04) | (0.04) | (0.03) | (0.04) |
| Constant | 2.401*** | 1.275*** | 2.183*** | 1.175*** |
|  | (0.13) | (0.14) | (0.19) | (0.14) |
|  |  |  |  |  |
| R2 | 0.153 | 0.180 | 0.147 | 0.185 |
| Adjusted R2 | 0.149 | 0.177 | 0.144 | 0.182 |
| N (individuals) | 9006 | 10103 | 9006 | 10103 |

Note: country fixed effects not reported

**Figure S4a: Robustness Figure 4a, based on model S4**

**Figure S4b: Robustness Figure 4b, based on model S4**
